# Supplementary material for: TNF-α Plus IL-1β Induces Opposite Regulation of Cx43 Hemichannels and Gap Junctions in Mesangial Cells through a RhoA/ROCK-Dependent Pathway
Source: Int J Mol Sci. 2022 Sep 3;23(17):10097. doi: 10.3390/ijms231710097 (PMC9456118; doi:10.3390/ijms231710097)
Supplement: Supplementary file 1 [file ijms-23-10097-s001.zip › ijms-1903483-supplementary.pdf]

SUPPLEMENTARY FIGURES:

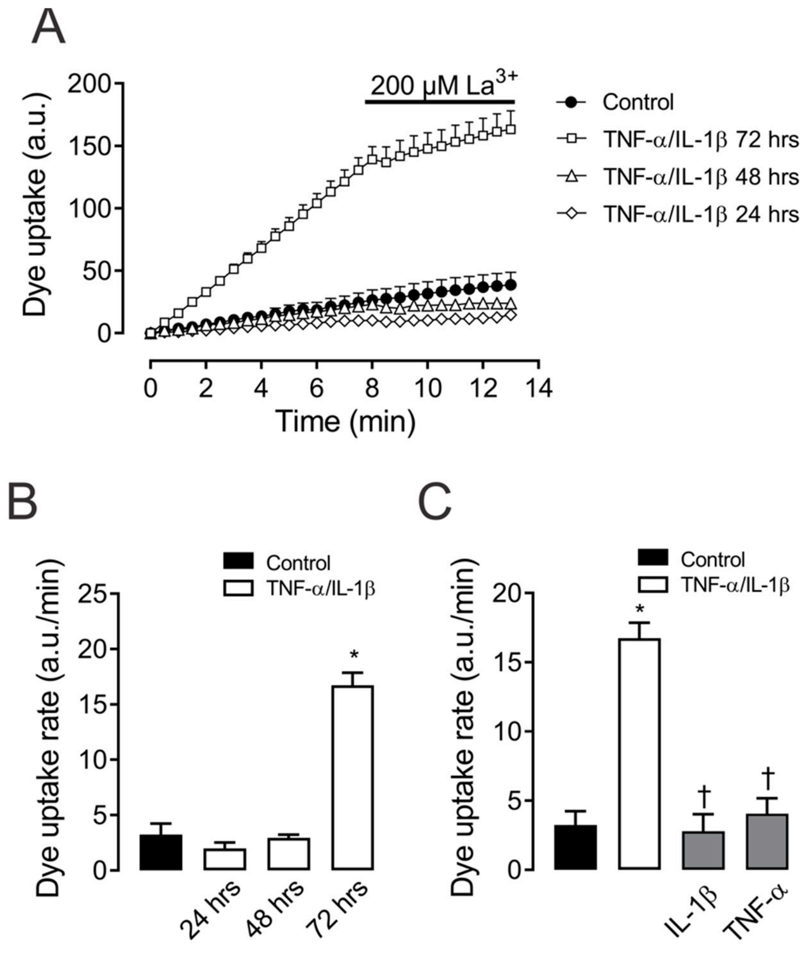

Supplementary Figure S1. TNF-α/IL-1β increases the ethidium uptake in mesangial cells. (**A**) Representative time-lapse recordings of 5 μM ethidium (Etd^+) uptake during 13 min in MES-13 cells under control conditions (black circles) or treated with TNF-α/IL-1β (10 ng/ml each) for different time periods (24-72 h). (**B**) Etd^+ uptake rate of MES-13 cells under control conditions (black bar) or after treatment with TNF-α/IL-1β for different time periods (24-72 h, white bar). (**C**) Etd^+ uptake rate of MES-13 cells under control conditions (black bar) or treated with TNF-α/IL-1β (white bar), TNF-α (10 ng/ml, dashed bar) or IL-1β (10 ng/ml, gray bar) for 72 h. In each experiment 15-25 cells were recorded. Each bar represents the mean value ± SEM of five independent experiments with three replicates each. For statistical analysis, each treatment was compared with its corresponding control, and significance was determined using a one-way ANOVA followed, in case of significance, by a Tukey post-hoc test. Statistical significance * $p < 0.05$ vs. Ctrl; † $p < 0.05$ vs. (TNF-α/IL-1β) 72 h.

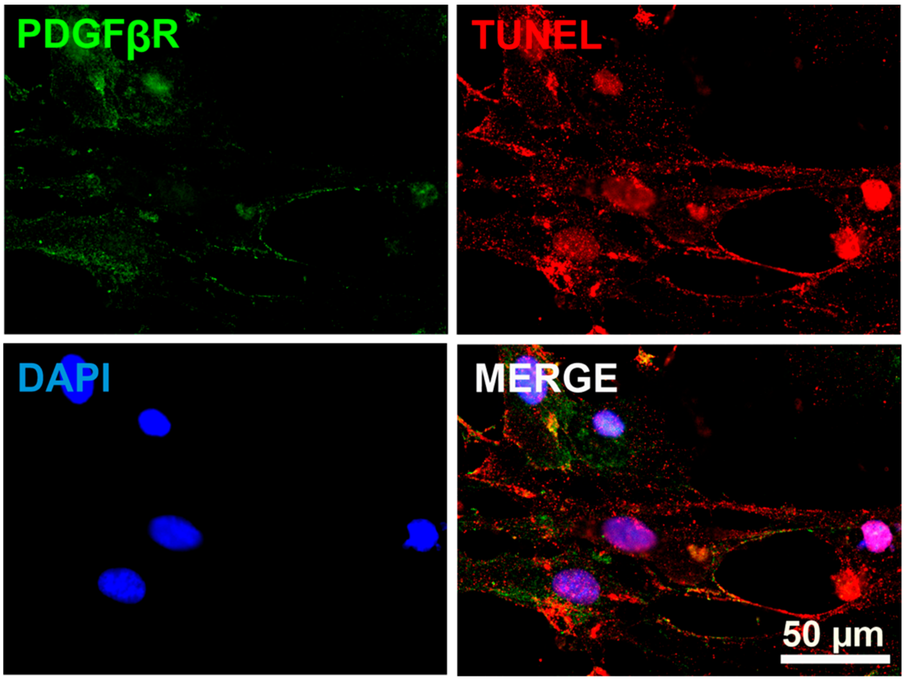

Supplementary Figure S2. Positive control of Tunel assay in primary mesangial cells. Fluorescence images depicting PDGFβR (green), Tunel (red) and DAPI (blue) staining by primary MCs stimulated with DNase I (generates strand breaks in the DNA to provide a positive TUNEL reaction).
